# Mapping Pharmacogenomic Determinants of Adverse Drug Reactions in Hypertension: A Systematic Review

**DOI:** 10.3390/ph19091488

**Published:** 2026-09-18

**Authors:** Viola Savy Dsouza, Jestina Rachel Kurian, Manoj Kalita, Kaustubh Bora, Sanjib Phukan, Rituparna Das, Ipsita Pal Bhowmick

**Affiliations:** 1 NIMS Institute of Public Health and Governance, NIMS University Rajasthan, Jaipur 303121, India; 2 Former Model Rural Health Research Unit Tripura, Agartala 799045, India; 3 Department of Applied Statistics and Data Science, Prasanna School of Public Health, Manipal Academy of Higher Education (MAHE), Manipal 576104, India; 4 ICMR-National Institute of Health Research (ICMR NIHR), Dibrugarh 786010, India; 5 Department of Community Medicine, Agartala Government Medical College, Agartala 799006, India; 6 Model Rural Health Research Unit Tripura, Agartala 799045, India

**Keywords:** hypertension, pharmacogenomics, adverse drug reactions, antihypertensive agents, personalized medicine, precision medicine, implementation science

## Abstract

**Objectives**: Adverse drug reactions (ADRs) to antihypertensive medications contribute to poor medication adherence and treatment discontinuation. Pharmacogenomic approaches offer a potential means of identifying genetic variants that predict ADR risks, thereby supporting more tailored antihypertensive therapy and improved tolerability. We systematically identify and synthesize available pharmacogenomic evidence for gene–drug–ADR interaction in hypertension across all major drug classes. **Methods**: This systematic review follows PRISMA guidelines. Five bibliographic databases were systematically searched for pharmacogenomic studies examining associations between genetic variants and adverse drug reactions in adults receiving antihypertensive therapy. Google Scholar was additionally used as a supplementary source during full-text retrieval and citation screening to identify potentially relevant peer-reviewed publications not captured by the primary database searches. Two reviewers independently screened studies, extracted data, and assessed methodological quality using the Newcastle–Ottawa Scale. The protocol was registered in PROSPERO (CRD420251247856). **Results**: Seven studies published between 2002 and 2024 fulfilled the inclusion criteria, encompassing populations from Pakistan, China, Russia, South Africa, Spain, South Korea, and Japan. Notably, no pharmacogenomic evidence was identified for beta-blockers, angiotensin receptor blockers, thiazide diuretics, alpha-blockers, or loop diuretics when used as antihypertensives. The studies focused on two antihypertensive medication classes: angiotensin-converting enzyme inhibitors (ACE-I) and calcium channel blockers (CCBs). Variants in BDKRB2 have significant links to ACE-I-induced cough in different populations, with individual investigations identifying other genes involved in bradykinin metabolism and signalling (MME, PTGER3) and hepatic drug transport (SLCO1B1). Associations between CCB-related peripheral edema and CYP3A5 polymorphisms were found in Chinese Han and Russian Caucasian groups. The quality of the studies varied, but two were rated as good. **Conclusions**: Preliminary pharmacogenomic evidence linking antihypertensive medications to ADRs is emerging but remains limited in scope and concentrated in two drug classes. These findings represent early-phase, hypothesis-generating contributions to the evidence base required for personalized medicine in hypertension pharmacotherapy. Realizing this potential will require prospective validation in diverse, multi-ethnic cohorts, standardization of ADR reporting and causality assessment, and sustained investment in pharmacogenomic implementation science.

## 1. Introduction

Hypertension is a multifactorial condition shaped by both biological and lifestyle factors, including physical inactivity, high salt and fat intake, alcohol, and tobacco use [1,2]. Its prevalence is rising worldwide, extending from high-income nations to emerging economies such as India, China, and several African countries, where rapid urbanization has intensified the public health burden [3]. Globally, an estimated 1.28 billion adults aged 30–79 years were living with hypertension in 2019, nearly double the 650 million reported in 1990 [4,5]. Furthermore, the 2021 Global Burden of Disease study highlighted a concerning 36.11% rise in hypertension-related disease burden among youth and young adults, reflecting a shift in risk to younger populations [6].

The emergence of pharmacogenomics as a foundational discipline within personalized medicine has created new opportunities for anticipating and mitigating adverse drug reactions (ADRs) through genotype-informed research approaches. In the management of hypertension (a condition requiring long-term, often lifelong pharmacotherapy), the potential for pharmacogenomically guided drug selection and dosing to reduce ADR burden represents a longer-term aspirational goal of personalized medicine, though one that remains substantially unrealized and contingent on considerably more evidence than is currently available.

Antihypertensive pharmacotherapy, commanding a global market valuation of USD 23.9–30.5 billion in 2024 with a projected expansion to USD 35.0–50.0 billion by 2033–2035 (CAGR 2.60–7.18%), is accompanied by a diverse array of ADRs that are contingent upon genetic, ethnic, and environmental determinants [7,8,9]. Clinically significant ADRs include dry cough and angioedema with angiotensin-converting enzyme inhibitors, peripheral edema and vasodilatory flushing with calcium channel antagonists, electrolyte perturbations and hyperuricemia with diuretic agents, and fatigue or bradyarrhythmias with β-adrenergic receptor antagonists [10]. A descriptive compilation of known ADRs to antihypertensive drugs is given in Table 1.

The prevalence and severity of these reactions are not uniform, but instead exhibit marked variation across drug classes, ancestral backgrounds, and age strata, underscoring the critical role of pharmacogenomic diversity in shaping therapeutic outcomes [17]. These ADRs are major factors driving poor medication adherence, which remains the principal barrier to achieving effective blood pressure control globally [18]. Patients often feel deterred from continuing to their therapy when side effects affect their quality of life or result in unpleasant symptoms [19]. In particular, these drugs were associated with nausea, constipation, palpitations, ankle or leg swelling, cold hands or feet, cramps, dry cough, skin rash, frequent urination, and diminished sexual desire. For instance, the likelihood of non-adherence was approximately 5.5 times higher in patients who experienced nausea than in those who did not. Similarly, ankle or leg swelling associated with nifedipine or amlodipine had an adjusted odds ratio (aOR) of about 2.26–2.22, which roughly doubled the odds of poor adherence in those who experienced this ADR [20]. Poor tolerability negatively affects drug compliance, which remains the principal barrier to effective hypertension control, and thereby diminishes the benefits of otherwise efficacious therapies [21].

Genetic polymorphisms in drug-metabolizing enzymes, transporters, and receptors, including CYP2D6, ACE I/D, SLC22A1, and NAT2, appear to modulate the risk and severity of ADR [22,23]. Yet the available evidence is fragmented and disproportionately derived from populations of European ancestry, which constrains its generalizability to the global population, particularly the global south [24,25]. Since allele frequencies and gene–drug interactions differ substantially across ethnic groups, the persistent underrepresentation of any group perpetuates inequities in precision hypertension care [26,27,28].

Despite the long-standing recognition of variability, and drug compliance issues due to ADRs, the systematic integration of pharmacogenomic insights into hypertension management remains limited [29,30]. The rate of adoption of pharmacogenomics in clinical settings has been limited by poor knowledge diffusion among physicians regarding the domain. Some studies reporting only 2% of clinicians aredemonstrating excellent perceived familiarity with the domain and its application [31]. Additionally, studies show that there is very little use of pharmacogenomic data with strong and high-confidence evidence because of obstacles like a lack of expertise and guidance for managing and interpreting data or making decisions [32].

In this context, evidence synthesis of the genetic factors contributes to ADRs as highlighted in the review articles and represents a necessary step towards building the evidence base that may, in the future, inform more rationally designed pharmacotherapy, provided that the associations identified are prospectively validated across diverse populations [33,34,35]. Integrating pharmacogenomics findings with sustained lifestyle modification offers a more holistic approach to hypertension management, shifting the focus from symptomatic control alone to broader promotion of wellness [36]. Evidence strongly suggests that lifestyle interventions like optimizing diet, physical activity, regulating weight, reducing stress, and managing sleep can lead to positive physiological changes at the cellular and systemic levels, possibly affecting inflammatory reactions, metabolic pathways, and epigenetic regulation [37]. Such approaches may increase drug tolerance, adherence, and support when paired with customized pharmacotherapy based on a person’s genetic and metabolic profile [38].

It is therefore helpful to map the genetic determinants of ADRs and determine the consistency of these gene–drug–ADR interactions across various worldwide populations and studies [39]. The available pharmacogenomic evidence for antihypertensive-related ADRs is sparse, many of which involve small, ethnically homogeneous populations. To the best of our knowledge, no systematic review has comprehensively examined gene–drug–adverse drug reaction interactions in hypertension. Our systematic review integrates available evidence from relevant sources and employs a structured quality-focused approach, with the aim of mapping pharmacogenomic determinants of ADRs in hypertension care. By synthesizing current evidence in a transparent and reproducible manner, this review focuses on supporting physicians and practitioners in informing their clinical practice. Such synthesis can guide the development of clinical guidelines, highlight gaps that warrant targeted investigation, and advance the application of pharmacogenomics in global hypertension care. This personalized medicine approach is an essential step to benefit the patients with hypertension to receive a treatment regimen that suits their genetic and metabolomic profiles, thereby improving outcomes, including compliance.

By synthesizing current evidence in a transparent and reproducible manner, this review seeks to provide a structured evidence map that may inform future research priorities and hypothesis-generating directions for the field. Such a synthesis can help identify gaps warranting targeted investigation and may, in the longer term, contribute to the evidence base needed to advance the application of pharmacogenomics in hypertension care, contingent on prospective validation and methodological standardization.

## 2. Materials and Methods

The article was written and reported as per the “Preferred Reporting Items for Systematic Reviews and Meta-Analyses (PRISMA)” checklist [39]. This review was conducted in accordance with a protocol that has been registered in PROSPERO (CRD420251247856).

### 2.1. Data Sources and Study Selection

Relevant studies were identified through systematic searches of five bibliographic databases: PubMed, Scopus, Embase, Web of Science, and Cochrane Library. In addition, Google Scholar was used as a supplementary search source during full-text retrieval and citation screening, with the aim of identifying potentially relevant peer-reviewed publications not captured by the primary database searches. Only peer-reviewed journal articles meeting all predefined eligibility criteria were considered for inclusion, regardless of the source through which they were identified. The initial database search was conducted in August, 2025 during the protocol-development phase. The final database search was conducted on 6 December 2025 without imposing any date restrictions. The authors devised the search strategy based on their prior experience in designing search approaches for similar research domains [40,41]. The employed search strategy is available in Supplementary Materials. The output from various databases was imported into Rayyan.ai to identify and remove duplicates. Title, abstract, and full-text screening was performed independently by two reviewers using predefined eligibility criteria. Studies were included only when consensus was achieved between both reviewers. Inter-rater reliability for full-text screening was evaluated using Cohen’s kappa statistic prior to resolving disagreements. Any discrepancies were settled through discussion. The study selection process is presented using a PRISMA 2020 flow diagram, with explicit documentation of exclusion reasons. The PRISMA 2020 checklist is available in Table S4 of the Supplementary Materials.

### 2.2. Inclusion Criteria

ADRs, as defined by the World Health Organization, “are responses to medicinal products that are noxious and unintended and occur at doses normally used in humans for prophylaxis, diagnosis, therapy of disease, or modification of physiological function.” [42] We included studies involving adult patients aged 18 years or older with hypertension undergoing antihypertensive therapy, where pharmacogenomic determinants of ADR were evaluated. Eligible studies included any pharmacogenomic investigations such as candidate gene analysis studies, genome-wide association studies (GWAS), exome sequencing, or related approaches, examining genetic variants (pharmacogenes) associated with ADR to antihypertensive medications. This review focuses on major classes of antihypertensive drugs, including beta-blockers, angiotensin-converting enzyme inhibitors, angiotensin receptor blockers, calcium channel blockers, adrenergic blockers, and diuretics. Primary research studies, namely randomized controlled trials, cohort studies, and case–control studies and pharmacogenomic association studies, conducted among hospitalized or non-hospitalized populations, irrespective of sex or geographical location were eligible for inclusion. Studies focusing on prescription errors, dosing errors, or non-ADR-related outcomes were excluded.

### 2.3. Exclusion Criteria

Studies enrolling pregnant or lactating women or participants younger than 18 years were excluded. Only English-language publications were considered. Secondary literature including narrative reviews, systematic reviews, editorials, commentaries, perspective or debate articles, and review protocols were also excluded. Grey literature, defined as research not published in peer-reviewed academic journals or not subjected to a formal peer-review process (e.g., preprints), was not included. Records identified through supplementary citation searching in Google Scholar were subject to the same eligibility criteria and were included only where they constituted peer-reviewed journal publications meeting all inclusion criteria; any non-peer-reviewed records identified through this route were excluded in the same manner as those from primary database searches. In addition, case reports and case series were excluded. Studies in which antihypertensive medications were prescribed for indications other than hypertension were also excluded.

### 2.4. Data Extraction, Synthesis, and Analysis

Independent data extraction was carried out by two reviewers using a predeveloped data extraction form. To ensure consistency, the form was piloted on three studies, during which discrepancies in interpretation were discussed and resolved, and the data extraction process was standardized. The extracted data comprised key study characteristics (including first author, year of publication, country and setting, study design, and sample size), participant characteristics (age, sex, population ancestry, and comorbidities, where reported), antihypertensive drugs studied and their respective drug classes, pharmacogenes and specific genetic variants investigated, and primary outcome measures, specifically the incidence and type of ADRs following antihypertensive treatment. Details on ADR definitions and detection methods were recorded, along with genetic association results. Where explicitly reported, causality assessment tools and dosage information were documented. Any disagreements between reviewers were resolved through discussion and consensus. Study characteristics and effect estimates describing the association between genetic polymorphisms and antihypertensive-related ADRs were comprehensively presented in tabular format. Findings were subsequently summarized according to an antihypertensive drug class. Due to substantial heterogeneity across studies and the limited number of primary studies examining the same polymorphism, a quantitative meta-analysis was not performed. In several instances, only a single study evaluated a specific genetic variant, precluding meaningful statistical pooling. Therefore, a structured qualitative synthesis was undertaken. Two reviewers collaboratively conducted the risk of bias assessment in accordance with the study design. Cohort and case–control studies were appraised using the Newcastle–Ottawa Scale [43]. Any differences in judgement were resolved through discussion and mutual agreement.

### 2.5. Variant Annotation and Allele Frequency Retrieval

For each pharmacogenomic variant identified in included studies, allele frequency annotation was attempted using publicly available population genetics databases, including the Genome Aggregation Database (gnomAD v4.1). Where variants were described with validated dbSNP reference identifiers (rsIDs), global and ancestry-stratified allele frequencies were extracted for the following population groups: global, European, South Asian, East Asian and African/African American.

## 3. Results

**Figure 1:**
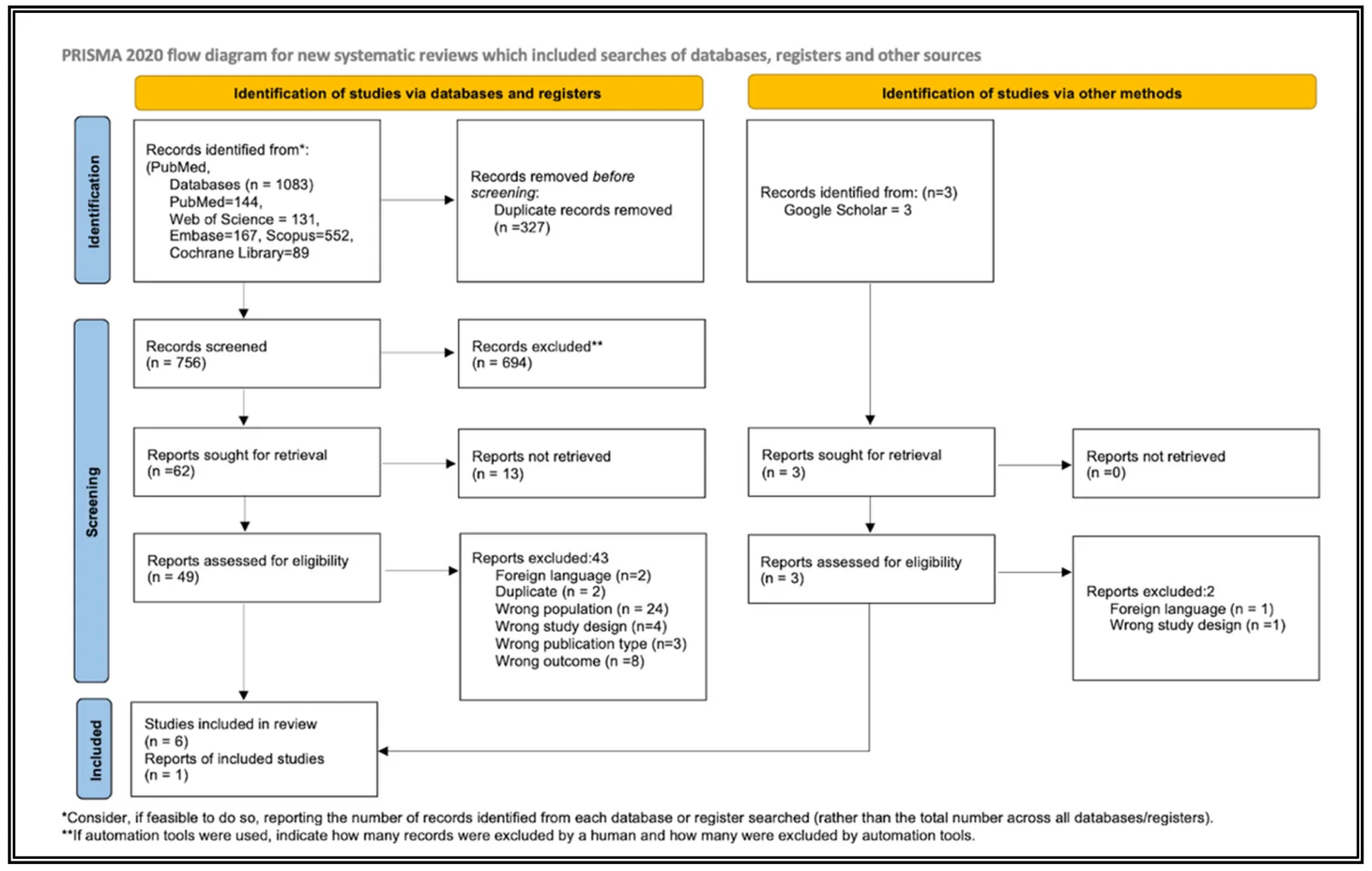
PRISMA 2020 flow diagram.

Database searches across five specified databases (PubMed, Web of Science, Embase, Scopus, and the Cochrane Library) yielded 1083 records; after removing 327 duplicates, 756 records underwent title and abstract screening, 62 reports were sought for full-text retrieval, of which 49 were retrieved and assessed for eligibility. Following full-text evaluation, 43 reports were excluded (Figure 1 and Supplementary Materials). Simultaneously, three additional records were identified through Google Scholar during full-text retrieval, of which one was included. Ultimately, seven studies met the inclusion criteria and were included in the synthesis. Inter-rater agreement for full-text screening was substantial (κ = 0.66), with an agreement percentage of 91.8% (*p* < 0.001).

The seven included studies were published between 2002 and 2024. The key characteristics of the included studies are summarized in Table 2. These studies were conducted across different settings of the world, including Pakistan, China, Russia, South Africa, Spain, South Korea, and Japan. Study populations represented diverse ethnic backgrounds, including South Asian (Punjabi, Pashtun, Kashmiri, Sindhi), Chinese Han, Japanese, Korean, Caucasian, and mixed-ancestry African populations [44,45,46,47,48,49,50]. Notably, no studies from sub-Saharan Africa (excluding the South African mixed-ancestry study), South Asia (excluding Pakistan), Latin America, or the Middle East were identified, representing a significant evidence gap. Regarding the study designs, five studies employed a case–control design, while two studies used a cohort design [44,45,46,47,48,49,50]. Recruitment was predominantly hospital-based, with some studies additionally recruiting participants from outpatient clinics. The majority of studies included individuals with essential or primary hypertension, while one study focused on arterial hypertension grades I–II [48]. Two antihypertensive drug classes were investigated: angiotensin-converting enzyme inhibitors (ACE inhibitors) and calcium channel blockers (CCBs). ACE inhibitor-induced cough was the most commonly reported ADR and was evaluated in studies conducted in Pakistan, South Africa, Spain, South Korea, and Japan. ADRs associated with CCBs, primarily peripheral or lower-extremity edema and skin flushing, were examined in studies from China and Russia.

Genetic investigations focused on a limited set of candidate genes with established biological relevance. SLCO1B1 was assessed in relation to ACE inhibitor-induced cough in a South Asian cohort [50]. CYP3A5 variants were evaluated in association with CCB-induced edema in both Chinese and Russian populations [48,49]. For ACE inhibitors-induced cough, multiple studies examined genes involved in bradykinin metabolism and signalling, including ACE, BDKRB2, and MME, while PTGER3 was reported as a protective factor in one European study [44]. Mean participant age ranged from the late 40s to early 60s, with cases generally younger than controls in case–control designs. Gender distribution varied by ADR, with a higher proportion of women among ACE inhibitor-induced cough, while CCB he pharmacokinetic role of CYP3A5 in influencing drug exposure and may help explain interindividual variability-associated edema studies showed more balanced or male-predominant populations. Comorbidities were reported in only one study (Moholisa et al.) [47] with diabetes mellitus affecting approximately 25–46% of ACE inhibitors-related ADR cases. Data collection methods included structured questionnaires, clinical examination, and medical record review, sometimes in combination, with one study applying WHO–UMC causality assessment criteria [49]. Based on NOS criteria, two studies were rated as good quality [45,50], three as fair quality [46,47,49], and two as poor quality [44,48]. The methodological quality of the included studies assessed using the Newcastle–Ottawa Scale is presented in Table 3.

Given the observed heterogeneity across studies in terms of study design, populations, antihypertensive drug classes, ADRs studied, and genetic variants assessed, results were synthesized according to drug class–ADR pairs and genetic pathways. Accordingly, the results are presented in two sections: (i) genetic associations with ACE inhibitor-induced cough, and (ii) genetic associations with calcium channel blocker-associated adverse reactions.

### 3.1. ACE Inhibitor-Induced Cough

#### 3.1.1. BDKRB2 (Bradykinin B2 Receptor)

Four case–control studies evaluated *BDKRB2* variants in relation to ACE inhibitor-induced cough [44,45,46,47]. In a Japanese population, Mukae et al. (2002) reported a strong association between the −58 T promoter allele and cough susceptibility (OR = 3.17, 95% CI 2.05–4.92; *p* < 0.001), with cough developing within two weeks of ACE inhibitor initiation [45]. Similarly, Moholisa et al. (2013) demonstrated that carriage of the −9 allele of the exon-1 insertion/deletion polymorphism was significantly associated with both ACE inhibitor-induced cough and angioedema (*p* = 0.008) [47]. In contrast, Woo et al. (2008) observed no significant associations between *BDKRB2* promoter or exon–intron junction polymorphisms and cough risk in a Korean cohort [46]. Grilo et al. (2011) further identified *BDKRB2 rs8012552* as significantly associated with an increased cough risk in a Spanish population (*p* = 0.012) [44].

#### 3.1.2. ACE (Angiotensin-Converting Enzyme)

Mukae et al. (2002) [45] and Woo et al. (2008) [46] found no overall association between the *ACE* insertion/deletion polymorphism and cough risk. Grilo et al. (2011) reported sex-specific effects, with increased risk observed in women and a protective effect in men [44].

#### 3.1.3. MME and PTGER3

In a large Spanish case–control study, Grilo et al. (2011) identified MME rs2016848 as significantly associated with increased susceptibility to ACE inhibitor-induced cough (*p* = 0.002). Conversely, PTGER3 rs11209716 demonstrated a protective association (*p* = 0.002) [44].

#### 3.1.4. SLCO1B1 (Drug Transport Pathway)

A prospective cohort study by Sheikh et al. (2024) evaluated six *SLCO1B1* variants using targeted sequencing in Pakistani patients receiving ACE inhibitors. Dry cough occurred in 51% of participants, typically within two weeks of treatment initiation [50]. Several missense, synonymous, and intronic variants, as well as haplotypes, were significantly associated with cough susceptibility after adjustment for clinical covariates, implicating altered hepatic drug transport as a modifier of ACE inhibitor-related adverse effects. The reported genetic variants associated with ACE inhibitor-induced cough are summarized in Table 4.

### 3.2. Calcium Channel Blocker-Associated Peripheral Edema

#### CYP3A5 (Drug Metabolism Pathway)

Two studies evaluated *CYP3A5* polymorphisms in relation to amlodipine-associated adverse reactions, primarily peripheral edema, in patients with hypertension [48,49]. In a hospital-based case–control study among Chinese Han patients, Liang et al. (2021) investigated three *CYP3A5* variants (*rs15524* [*1D], *rs4646453* [*1E], and *rs776746* [3]) using targeted next-generation sequencing. Peripheral edema occurred in 26.7% (64/240) of patients treated with amlodipine or L-amlodipine for at least four weeks. Minor allele frequencies for all three variants were lower among edema cases than controls, and both single-variant and haplotype analyses demonstrated significant associations with edema susceptibility after adjustment for demographic and clinical covariates. ADR were classified using WHO–UMC causality assessment, supporting a probable or possible causal relationship with amlodipine exposure. In a prospective cohort study conducted in a Caucasian Russian population, Sychev et al. (2019) examined the *CYP3A5 A6986G* polymorphism using real-time PCR in patients receiving amlodipine at doses of 5–10 mg/day for 12 weeks. Lower-extremity edema was observed in 44% of heterozygous AG carriers, compared with 0% among GG homozygotes, while skin flushing was more frequent in GG carriers. Genetic associations identified for calcium channel blocker-related adverse reactions are presented in Table 5.

Ancestry-stratified population allele frequencies for pharmacogenomic variants identified in this review are presented in Supplementary Materials, Table S3. For variants with validated dbSNP rsIDs, allele frequency data were retrieved from the Genome Aggregation Database (gnomAD). Substantial cross-ancestry variability was observed for several variants. For example, *CYP3A5* rs15524 and rs4646453 demonstrated notably higher allele frequencies in African, South Asian, and East Asian populations compared with European populations. Similarly, the *CYP3A5* rs776746 variant showed marked ancestry-specific variation, with lower allele frequency in African populations (0.29) relative to European (0.93), South Asian (0.72), and East Asian (0.73) populations.

Variability across ancestral groups was also observed for variants involved in bradykinin signalling pathways, including *BDKRB2* rs8012552, *MME* rs2016848, and *PTGER3* rs11209716. Such differences in allele distribution may partly contribute to variability in antihypertensive ADR susceptibility across populations and may limit the generalizability of findings derived predominantly from single-ancestry cohorts. These observations further reinforce the importance of conducting larger multi-ethnic pharmacogenomic studies, particularly in underrepresented Global South populations, to support more equitable and evidence-informed personalized medicine approaches in hypertension.

## 4. Discussion

This systematic review identified seven eligible studies examining pharmacogenomic determinants of ADRs associated with antihypertensive therapy. Although antihypertensive pharmacotherapy encompasses multiple drug classes in routine clinical practice, the available pharmacogenomic evidence was limited in scope and primarily focused on two classes: angiotensin converting enzyme inhibitors and calcium channel blockers [51].

ACE-I, CCB’s, and thiazide diuretics are widely recommended as first-line therapies for the management of hypertension and are associated with clinically significant ADR. ACE-I are commonly associated with dry cough (10–20%), angioedema (1.2–1.9%), hyperkalemia (2–6%), and renal dysfunction (2–11%) [51]. Amlodipine, a commonly prescribed calcium channel blocker, is frequently linked to peripheral edema, affecting approximately 10.7% of users and contributing to treatment withdrawal rates of about 2.1% [52].

Ancestry, sex and gender exert profound influences on drug response and susceptibility to adverse effects. Variations in endocrine regulation, body composition, and renal electrolyte dynamics converge with gender-specific patterns [53]. Women, for example, have a higher incidence of cough and angioedema in response to ACE-Is, whereas men exhibit greater vulnerability to diuretic-induced metabolic disturbances of adherence and polypharmacy to generate distinct risk architectures [53,54]. Considering diversity across biological, behavioural, and demographic dimensions is therefore essential for understanding the genetic basis of ADR and for advancing a more equitable framework of precision hypertension care [55]. In this context, systematic synthesis of available pharmacogenomic evidence provides an important foundation for identifying gaps and directing future investigative priorities.

Our systematic review identifies two different genetic pathways that may be involved in antihypertensive ADRs; the drug metabolism pathway (CYP3A5) in calcium channel blocker-associated edema and the bradykinin pathway (BDKRB2, ACE, MME) in angiotensin-converting enzyme inhibitor-induced cough.

CYP3A5 is the primary enzyme metabolizing amlodipine and other dihydropyridine CCBs. For amlodipine-associated peripheral edema, evidence from both Liang et al. (2021) and Sychev et al. (2019) supports a role for CYP3A5 polymorphisms based on data from Chinese Han and Russian Caucasian populations, respectively [48,49]. These findings are consistent with the pharmacokinetic role of CYP3A5 in influencing drug exposure and may help explain inter-individual variability in susceptibility to vasodilatory adverse effects. Liang et al. applied structured causality assessment using the WHO–UMC scale, providing greater confidence in the attribution of edema to amlodipine exposure compared with earlier observational approaches [49,56]. However, comparability between studies was limited. Different genetic variants were examined, including rs776746, rs15524, and rs4646453 in the Chinese study, and the A6986G polymorphism in the Russian cohort. Collectively, the evidence from Liang et al. and Sychev et al. suggests that drug metabolism gene CYP3A5, rather than target receptor pathways, appears to be likely to have a role in ADRs caused by calcium channel blockers. However, the small number of studies and lack of methodological standardization highlight arising from the combined effects of impaired bradykinin the need for additional studies to determine the clinical significance of CYP3A5 variants.

In some populations, including Japanese, South African, and Spanish cohorts, variations in the BDKRB2 gene were most consistently linked to ACE-I-induced cough; one negative result was found in a Korean group. Grilo et al. (2011) reported sex-specific effects, with increased susceptibility in women and a protective effect in men [44]. The study also suggested genetic variation in ACE and MME influences the extent of bradykinin degradation. Genetic variants within the bradykinin pathway emerged as the most consistently implicated contributors to ACE inhibitor-induced cough rising from the combined effects of impaired bradykinin degradation, enhanced receptor level responsiveness, and downstream modulation of sensory nerve activation [57]. The observed consistency of associations is likely at the pathway level, thereby explaining heterogeneity in individual variants and population-specific effects [58]. Studies examined different BDKRB2 genetic variants across populations, including promoter variants, insertion deletion variants, and rs8012552, which made a direct comparison between studies difficult and prevented the identification of a standard testing approach. As a result, the ability of BDKRB2 testing to reliably predict ACE inhibitor-induced cough remains uncertain.

The purpose of this review is to gather and summarize pharmacogenomic evidence tying antihypertensive medications to specific genes, ADR, and underlying biological pathways. However, the available literature was limited in scope and exhibited significant diversity in study design, populations studied, genetic variations evaluated, and outcome definitions. This narrow body of evidence is consistent with broader field findings. According to Zhang et al.’s latest bibliometric analysis, hypertension remains a relatively peripheral and underdeveloped area of pharmacogenomics research, despite its significant global disease burden [59]. A further system-level constraint observed in this review is the under-representation of populations with the highest burden of hypertension in pharmacogenomic studies [60]. While hypertension prevalence and related complications are rising most rapidly in low- and middle-income countries, (particularly in South Asia and Sub-Saharan Africa), pharmacogenomic investigations of antihypertensive-related ADRs remain limited in these settings [61]. Notably, several of these regions also face challenges related to medication adherence and variability in treatment response, further amplifying the clinical importance of such research. India, including its north-eastern states where hypertension burden and treatment disparities are increasingly recognized, represents a notable example of this evidence gap [62]. Similar under-representation is evident across other high-burden global settings. No pharmacogenomic evidence was identified for beta-blockers, angiotensin receptor blockers, thiazide diuretics, alpha-blockers, or loop diuretics, representing a substantial evidence gap. Addressing this disparity through well-designed, multi-ethnic pharmacogenomic studies is essential to ensure equitable advancement of precision hypertension care and to prevent widening global health inequities. This review should therefore be read as a systematic evidence map of reported pharmacogenomic associations in antihypertensive therapy, one that identifies research priorities and highlights gaps in the existing literature, rather than as a source of clinically directive genotype or allele-level recommendations.

At present, the pharmacogenomics of antihypertensive medications in relation to ADRs is some distance away from translation and integration into standard clinical decision-support tools. Progress in this area requires coordinated multi-ethnic research initiatives to better understand population-specific genetic effects, particularly in areas with mounting hypertension burden. Further research is also required to address currently understudied medication classes, such as beta blockers, thiazide diuretics, and angiotensin receptor blockers, which are routinely used in clinical practice. Furthermore, validation of the reported relationships between BDKRB2 and CYP3A5 in larger, prospective cohorts is required to determine their clinical significance. Finally, implementation studies comparing genotype-guided prescribing with standard care will be necessary to assess whether pharmacogenomic information may meaningfully improve tolerance and treatment results in hypertension.

The integration of pharmacogenomics into personalized medicine strategies for hypertension management represents an evolving translational objective. The pharmacogenomic associations identified in this review, particularly involving genes related to bradykinin signalling (*BDKRB2*, *MME*), drug metabolism (*CYP3A5*), and drug transport (*SLCO1B1*), suggest that a proportion of interindividual variability in antihypertensive ADR susceptibility may be genetically mediated. However, the current evidence base remains limited, and most identified associations are not currently supported by CPIC clinical practice guidelines specific to antihypertensive ADR prediction. Furthermore, available PharmGKB/ClinPGx clinical annotation evidence levels for several reported variants remain within lower evidence categories, reflecting the preliminary nature of the available data. The translational pathway from exploratory pharmacogenomic association to clinically actionable personalized medicine recommendation has been successfully demonstrated in other therapeutic areas, including *CYP2C9/VKORC1*-guided warfarin dosing and *CYP3A5*-guided tacrolimus therapy. Similar progress in antihypertensive pharmacogenomics will likely require large prospective multi-ethnic studies, harmonized ADR phenotyping approaches, replication across diverse populations, and implementation-focused research evaluating the clinical utility of genotype-guided prescribing strategies. Readers seeking deeper pharmacogenomic characterization of individual variants identified in this review are directed to ClinPGx (formerly PharmGKB) as an authoritative resource for genotypic- and allelic-level evidence classification. Systematic integration of ClinPGx annotations into future updates of this evidence map would be a valuable next step, contingent on the availability of a more robust and replicable primary evidence base.

Even if future research were to generate sufficiently robust pharmacogenomic evidence in this area, the translation of such findings into personalized medicine approaches for hypertension management would face substantial implementation barriers. Clinician familiarity with pharmacogenomics remains variable, and many physicians and pharmacists may lack confidence in interpreting genotype-guided prescribing recommendations or using resources such as CPIC. In addition, infrastructure required for pharmacogenomic-guided prescribing including accredited genotyping facilities, timely testing pathways, and electronic health record-integrated clinical decision-support systems remains limited in many healthcare settings. Cost, limited reimbursement frameworks, and the lack of pharmacoeconomic evidence for genotype-guided antihypertensive prescribing also represent important barriers to implementation. Regulatory guidance specific to pharmacogenomic-guided antihypertensive prescribing is also lacking. These considerations imply that even well-validated pharmacogenomic associations would require dedicated implementation science before they could meaningfully inform clinical practice.

Limitations of this review should be acknowledged, such as the substantial heterogeneity in ADR reporting and causality assessment across the seven included studies which prevented meaningful comparison across studies. Quantitative synthesis was not possible owing to substantial differences across studies in methodology, units of analysis, study populations, and outcome definitions. The current state of ADR reporting in the field for causality assessment and severity grading was inconsistent across studies, ranging from structured tools such as the Naranjo scale and WHO–UMC criteria to non-standard approaches. By limiting inclusion to hypertension-specific study populations, we excluded pharmacogenomic evidence from studies conducted in other clinical contexts, despite potential overlap in medications, ADR, and genetic mechanisms. This decision allowed the synthesis to focus on the interpretability and clinical relevance of the findings for hypertension care alone and identified the gaps. In addition, the review was limited to English-language publications, which may have introduced language bias and affected generalisability. Furthermore, comorbidities, sex-specific effects, and gene–environment interactions were rarely examined, further constraining interpretation. Collectively, these limitations underline the need for larger, methodologically harmonized, multi-ethnic primary studies to generate reproducible effect estimates and enable robust future quantitative synthesis. Greater standardization of ADR ascertainment, severity grading, and causality assessment will be important for generating more robust and comparable pharmacogenomic evidence in antihypertensive therapy. Future studies should prospectively adopt harmonized reporting approaches to improve cross-study comparability and facilitate future quantitative synthesis.

The underrepresentation of Global South populations in pharmacogenomic studies of antihypertensive ADRs represents an important limitation of the current evidence base. Most included studies were conducted in East Asian or European populations, with minimal representation from African, South Asian, and Latin American settings despite the substantial hypertension burden in these regions. Given that pharmacogenomically relevant allele frequencies differ across ancestral groups, findings from European or East Asian cohorts may not be directly generalizable to other populations. Future research should prioritize large multi-ethnic prospective studies with standardized ADR definitions, harmonized causality assessment approaches, and broader inclusion of underrepresented populations. Greater use of genome-wide and multi-omics approaches, alongside implementation-focused pharmacogenomic studies evaluating genotype-guided prescribing, may help strengthen the evidence base required for equitable personalized hypertension care. Future research should also prioritize prospective pharmacogenomic studies in underrepresented Global South populations, incorporating standardized ADR definitions and causality assessment protocols, to build the evidence base needed for an equitable and meaningful implementation framework for precision hypertension care.

## 5. Conclusions

This comprehensive review summarized current pharmacogenomic evidence regarding gene–drug–ADR relationships in antihypertensive therapy, highlighting both the emerging potential of the field and its present limitations. Only seven eligible studies were identified, with most evidence focused on ACE inhibitor-associated cough and calcium channel blocker-related peripheral edema. Preliminary associations involving *BDKRB2* and *CYP3A5* suggest that interindividual genetic variability may contribute to susceptibility to antihypertensive-related ADRs. These associations are preliminary and hypothesis-generating in nature. Their potential relevance to personalized medicine approaches in hypertension management will depend on prospective validation in larger, diverse cohorts before any clinical application could be considered.

Several antihypertensive medication classes remain unexplored from a pharmacogenomic standpoint, and populations bearing the greatest burden of hypertension remain substantially underrepresented in current research. The present evidence base remains in the early discovery stage and is insufficient to support routine clinical implementation or genotype-guided prescribing. Nonetheless, this review provides a structured synthesis of existing evidence, identifies important evidence gaps, and highlights priorities for future research. Addressing these limitations through inclusive, methodologically rigorous, and well-powered primary studies will be necessary before pharmacogenomic findings in this area can move beyond hypothesis generation.

## Figures and Tables

**Table 1:** Key ADRs associated with commonly used antihypertensive agents.

Drug Class	Representative Agents	Primary Organ System Affected	Mechanism Related to ADRs	Clinically Significant ADR
Angiotensin converting enzyme inhibitors [11]	Enalapril, Lisinopril, Ramipril, Captopril	Respiratory, Integumentary, Vascular	Bradykinin accumulation due to ACE inhibition	Dry persistent cough, angioedema, hypotension, hyperkalemia
Angiotensin II receptor blockers [12]	Losartan, Valsartan, Telmisartan	Cardiovascular(CVS), Renal (Rare)	Angiotensin II receptor blockade	Dizziness, hypotension, hyperkalemia, rare angioedema and rare renal failure
Calcium channel blockers (dihydropyridines) [13]	Amlodipine, Nifedipine	CVS, Central nervous system (CNS) Peripheral vascular	Peripheral arteriolar vasodilation	Peripheral edema, vasodilatory flushing, headache, palpitations, dizziness
Calcium channel blockers (non-dihydropyridines) [13]	Verapamil, Diltiazem	Cardiovascular, Gastrointestinal, CNS	Reduced myocardial contractility and atrioventricular conduction	Bradycardia, atrioventricular block, constipation
Thiazide and thiazide-like diuretics [14]	Hydrochlorothiazide, Chlorthalidone	Renal, Metabolic, Electrolyte	Renal sodium and water excretion	Hypokalemia, hyponatremia, hyperuricemia, hyperglycemia
Loop diuretics [14]	Furosemide, Bumetanide	Renal, Electrolyte, Auditory, CNS	Inhibition of sodium reabsorption in loop of Henle	Electrolyte imbalance, dehydration, ototoxicity at high doses, Lithium toxicity
Beta adrenergic receptor antagonists [15]	Atenolol, Metoprolol, Propranolol	CVS, Respiratory, CNS, Metabolic	Reduced sympathetic stimulation	Fatigue, bradyarrhythmia’s, exercise intolerance, bronchospasm, Micturition disorders
Alpha adrenergic blockers [16]	Prazosin, Doxazosin	Cardiovascular, Autonomic nervous system, CNS	Peripheral vasodilation via alpha blockade	Postural hypotension, dizziness, reflex tachycardia, Syncope

**Table 2:** Characteristics of included pharmacogenomic studies examining antihypertensive drug-related adverse drug reactions.

Author, Year, Country	Ethnicity/Race	Study Design	Recruitment Setting	Type of HTN	Drug Class	ADR Type	Gene	ADR Assessment
Sheikh, 2024, Pakistan [50]	Punjabi, Pashtuns, Kashmiris, and Sindhis	Cohort	Clinic	Essential hypertension	ACE inhibitors	Dry cough	SLCO1B1	Dry cough is defined by symptom onset after ACEI initiation with resolution within 15 days of drug withdrawal (positive de-challenge)
Liang, 2021, China [49]	Chinese Han	Case–control	Hospital	Essential hypertension	Calcium channel blocker	Peripheral edema	CYP3A5	Peripheral edema assessed by two physicians using WHO–UMC causality scale (certain/probable/possible)
Sychev, 2019, Russia [48]	Caucasian race	Cohort	Hospital	Arterial hypertension (AH) I–II degree	Calcium channel blocker	Lower-extremity edema; skin flushing	CYP3A5	No formal causality assessment reported
Moholisa, 2013, South Africa [47]	Cape mixed ancestry/Black/White (self-identified)	Case–control	Hospital/ clinic	Hypertension	ACE inhibitors	Cough	ACE; BDKRB2 (bradykinin B2 receptor)	ADR defined by clinical occurrence with symptom resolution after ACEI withdrawal (positive de-challenge)
Grilo, 2011, Spain [44]	not specified	Case–control	Hospital	Essential hypertension	ACE inhibitors	ACE inhibitor-induced cough	MME and BDKRB2 (increased risk), PTGER3 (protective)	ACEI-induced cough identified by temporal association with treatment and improvement after withdrawal (positive de-challenge implied)
Woo, 2008, South Korea [46]	Koreans	Case–control	Hospital	Primary hypertension	ACE inhibitors	Dry cough (ACE inhibitor-induced coughing)	ACE, BDKRB2 promoter −58T/C SNP, BDKRB2 exon–intron junction −59C/A SNP	No formal causality assessment reported
Mukae, 2002, Japan [45]	Japanese	Case–control	Hospital outpatient clinics	Essential hypertension	ACE inhibitors	ACEI-induced dry cough	BDKRB2 (Bradykinin B2 receptor)	Dry cough developing within 2 weeks of ACEI initiation and resolving after withdrawal (positive de-challenge)

**Table 3:** Quality assessment of included studies using the Newcastle–Ottawa Scale.

Study	Selection	Comparability	Outcome/Exposure	Overall Quality
Study Design: Case–Control				
Mukae 2002 [45]	★ ★ ★	★	★ ★	Good
Liang 2021 [49]	★ ★	★	★ ★	Fair
Moholisa 2013 [47]	★ ★	★	★ ★	Fair
Woo 2008 [46]	★ ★	★	★ ★	Fair
Grilo 2011 [44]	★ ★	0	★ ★	Poor
Study Design: Cohort				
Sychev 2019 [48]	★ ★ ★	0	★	Poor
Sheikh 2024 [50]	★ ★ ★ ★	★ ★	★ ★	Good

**Note:** Stars (★) represent points awarded on the Newcastle–Ottawa Scale (maximum 9 points). Good quality: 7–9 stars; Fair quality: 4–6 stars; Poor quality: 0–3 stars.

**Table 4:** Genetic variants associated with ACE inhibitor-induced cough.

Gene	Pathway	Variant/Polymorphism	Study (Year)	Population	ADR	Genetic Model	Effect Direction
BDKRB2	Bradykinin signalling	−58 T/C	Mukae 2002 [45]	Japanese	Cough	Allelic	↑ Risk
BDKRB2	Bradykinin signalling	−9/+9	Moholisa 2013 [47]	South African	Cough/AE	Dominant	↑ Risk
BDKRB2	Bradykinin signalling	rs8012552	Grilo 2011 [44]	Spanish	Cough	Additive	↑ Risk
ACE	RAAS	I/D polymorphism	Grilo 2011 [44]	Spanish	Cough	Sex-stratified	↑ Risk (♀), ↓ Risk (♂)
MME	Bradykinin degradation	rs2016848	Grilo 2011 [44]	Spanish	Cough	Additive	↑ Risk
PTGER3	Prostaglandin	rs11209716	Grilo 2011 [44]	Spanish	Cough	Additive	↓ Risk
SLCO1B1	Drug transport	Multiple SNPs/haplotypes	Sheikh 2024 [50]	Pakistani	Cough	SNP/haplotype	↑ Risk

**Note: ↑** Increased risk; **↓** Decreased risk/protective effect; ♀ indicates female; ♂ indicates male.

**Table 5:** Genetic variants associated with calcium channel blocker-related adverse reactions.

Gene	Variant (rsID/Polymorphism)	Drug	ADR Phenotype	Author, Year	Design	Genetic Model	Effect Direction	Population
CYP3A5	rs776746 (*3)	Amlodipine	Peripheral edema	Liang, 2021 [49]	Case–control	Allelic/haplotype	↓ Risk (protective)	Chinese Han
CYP3A5	rs15524 (*1D)	Amlodipine	Peripheral edema	Liang, 2021 [49]	Case–control	Allelic	↓ Risk (protective)	Chinese Han
CYP3A5	rs4646453 (*1E)	Amlodipine	Peripheral edema	Liang, 2021 [49]	Case–control	Allelic/haplotype	↓ Risk (protective)	Chinese Han
CYP3A5	A6986G	Amlodipine	Edema/flushing	Sychev, 2019 [48]	Prospective cohort	Genotype comparison	↑ Risk	Caucasian (Russia)

**Note: ↑** Increased risk; **↓** Decreased risk/protective effect.

## Data Availability

No new data were created or analyzed in this study. Data sharing is not applicable to this article.

## Supplementary Materials

The following supporting information can be downloaded at: https://www.mdpi.com/article/10.3390/ph19091488/s1. Table S1: Keywords used for development of search queries; Table S2: Search queries (by database); Table S3: Ancestry-Stratified Population Allele Frequencies (AF); Table S4: PRISMA 2020 checklist. From the reference [63].
