# Supplementary material for: Mapping Pharmacogenomic Determinants of Adverse Drug Reactions in Hypertension: A Systematic Review
Source: Pharmaceuticals (Basel). 2026 Sep 18;19(9):1488. doi: 10.3390/ph19091488 (PMC13610982; doi:10.3390/ph19091488)
Supplement: Supplementary file 1 [file pharmaceuticals-19-01488-s001.zip › pharmaceuticals-4271883-supplementary.pdf]

Supplementary Materials

Table S1: Keywords used for development of search queries.

Category	Controlled Vocabulary (MeSH / Emtree)	Free-Text / Keywords
Hypertension / Population	"Hypertension"[Mesh]; "Essential Hypertension"[Mesh]	hypertension, “high blood pressure”, hypertensive
Antihypertensive Medications	"Antihypertensive Agents"[Mesh]; "Angiotensin-Converting Enzyme Inhibitors"[Mesh]; "Angiotensin Receptor Antagonists"[Mesh]; "Adrenergic beta-Antagonists"[Mesh]; "Calcium Channel Blockers"[Mesh]; "Diuretics"[Mesh]	antihypertensive*, “blood pressure medication*”, ACE inhibitor*, ARB*, “angiotensin receptor blocker*”, beta blocker*, “calcium channel blocker*”, “adrenergic blocker*”, alpha blocker*, diuretic*,"Angiotensin-converting enzyme inhibitor*","Adrenergic blocker*"
Pharmacogenomics / Genetic Determinants	"Pharmacogenetics"[Mesh]; "Pharmacogenomic Variants"[Mesh]	pharmacogenomic*, pharmacogenetic*, pharmacogene*, “genetic polymorphism”, “genetic variant”, “gene–drug interaction”, “drug response gene”, “pharmacokinetic polymorphism”, “pharmacodynamic variant”
Adverse Drug Reactions (ADRs)	"Drug-Related Side Effects and Adverse Reactions"[Mesh]; "Drug Toxicity"[Mesh]	("adverse drug reaction*" OR ADR OR "adverse drug event*" OR ADE OR "adverse drug effect*" OR "side effect*" OR "adverse effect*" OR "drug toxicity" OR "treatment intolerance" OR pharmacovigilance OR "drug-related side effect*" OR "drug related adverse reaction*" OR "medicine side effect" OR "ADR prediction" OR "adverse drug reaction prediction" OR "adverse outcome*" OR "undesirable effect*" OR "undesirable event*" OR "treatment emergent" OR "Drug complication*" OR "high risk medication*" OR "high risk drug*")

Table S2: Search queries (by database)

Query for PubMed (144)	
	((("Pharmacogenetics"[Mesh] OR "Pharmacogenomic Variants"[Mesh] OR pharmacogenomic* OR pharmacogenetic* OR pharmacogene* OR "genetic polymorphism" OR "genetic variant" OR "gene-drug interaction" OR "drug response gene" OR "pharmacokinetic polymorphism" OR "pharmacodynamic variant")) AND (("Drug-Related Side Effects and Adverse Reactions"[Mesh] OR "Drug Toxicity"[Mesh] OR "adverse drug reaction*" OR ADR OR "adverse drug event*" OR ADE OR "adverse drug effect*" OR "side effect*" OR "adverse effect*" OR "drug toxicity" OR "treatment intolerance" OR pharmacovigilance OR "drug-related side effect*" OR "drug related adverse reaction*" OR "medicine side effect" OR "ADR prediction" OR "adverse drug reaction prediction" OR "adverse outcome*" OR "undesirable effect*" OR "undesirable event*" OR "treatment emergent" OR "drug complication*" OR "high risk medication*" OR "high risk drug*"))) AND (("Antihypertensive Agents"[Mesh] OR "Angiotensin-Converting Enzyme Inhibitors"[Mesh] OR "Angiotensin Receptor Antagonists"[Mesh] OR "Adrenergic beta-Antagonists"[Mesh] OR "Calcium Channel Blockers"[Mesh] OR "Diuretics"[Mesh] OR antihypertensive* OR "blood pressure medication*" OR "ACE inhibitor*"

	OR ARB* OR "angiotensin receptor blocker*" OR "beta blocker*" OR "calcium channel blocker*" OR "adrenergic blocker*" OR "alpha blocker*" OR diuretic* OR "angiotensin-converting enzyme inhibitor*"))

Query for Web of Science (131)	
Search terms	(pharmacogenomic* OR pharmacogenetic* OR pharmacogene* OR "genetic polymorphism" OR "genetic variant" OR "gene-drug interaction" OR "drug response gene" OR "pharmacokinetic polymorphism" OR "pharmacodynamic variant") AND ("adverse drug reaction*" OR ADR OR "adverse drug event*" OR ADE OR "adverse drug effect*" OR "side effect*" OR "adverse effect*" OR "drug toxicity" OR "treatment intolerance" OR pharmacovigilance OR "drug-related side effect*" OR "drug related adverse reaction*" OR "medicine side effect" OR "ADR prediction" OR "adverse drug reaction prediction" OR "adverse outcome*" OR "undesirable effect*" OR "undesirable event*" OR "treatment emergent" OR "drug complication*" OR "high risk medication*" OR "high risk drug*") AND (antihypertensive* OR "blood pressure medication*" OR "ACE inhibitor*" OR ARB* OR "angiotensin receptor blocker*" OR "beta blocker*" OR "calcium channel blocker*" OR "adrenergic blocker*" OR "alpha blocker*" OR diuretic* OR "angiotensin-converting enzyme inhibitor*" OR "adrenergic blocker*")

Query for Scopus (552)	
Search terms	(TITLE-ABS-KEY (pharmacogenomic* OR pharmacogenetic* OR pharmacogene* OR "genetic polymorphism" OR "genetic variant" OR "gene-drug interaction" OR "drug response gene" OR "pharmacokinetic polymorphism" OR "pharmacodynamic variant")) AND (TITLE-ABS-KEY ("adverse drug reaction*" OR ADR OR "adverse drug event*" OR ADE OR "adverse drug effect*" OR "side effect*" OR "adverse effect*" OR "drug toxicity" OR "treatment intolerance" OR pharmacovigilance OR "drug-related side effect*" OR "drug related adverse reaction*" OR "medicine side effect" OR "ADR prediction" OR "adverse drug reaction prediction" OR "adverse outcome*" OR "undesirable effect*" OR "undesirable event*" OR "treatment emergent" OR "drug complication*" OR "high risk medication*" OR "high risk drug*")) AND (TITLE-ABS-KEY(antihypertensive* OR "blood pressure medication*" OR "ACE inhibitor*" OR ARB* OR "angiotensin receptor blocker*" OR "beta blocker*" OR "calcium channel blocker*" OR "adrenergic blocker*" OR "alpha blocker*" OR diuretic* OR "angiotensin-converting enzyme inhibitor*" OR "adrenergic blocker*"))

Query for Embase (167)	
	(pharmacogenomic*:ti,ab,kw OR pharmacogenetic*:ti,ab,kw OR pharmacogene*:ti,ab,kw OR 'genetic polymorphism':ti,ab,kw OR 'genetic variant': ti,ab,kw OR 'gene-drug interaction': ti,ab,kw OR 'drug response gene':ti,ab,kw OR 'pharmacokinetic polymorphism':ti,ab,kw OR 'pharmacodynamic variant': ti,ab,kw) AND ('adverse drug reaction*':ti,ab,kw OR adr: ti,ab,kw OR 'adverse drug event*':ti,ab,kw OR ade: ti,ab,kw OR 'adverse drug effect*':ti,ab,kw OR 'side effect*':ti,ab,kw OR 'adverse effect*':ti,ab,kw OR 'drug toxicity': ti,ab,kw OR 'treatment intolerance': ti,ab,kw OR pharmacovigilance:ti,ab,kw OR 'drug-related side effect*':ti,ab,kw OR 'drug related adverse reaction*':ti,ab,kw OR 'medicine side effect':ti,ab,kw OR 'adr prediction':ti,ab,kw OR 'adverse drug reaction prediction':ti,ab,kw OR 'adverse outcome*':ti,ab,kw OR 'undesirable effect*':ti,ab,kw OR 'undesirable event*':ti,ab,kw OR 'treatment emergent': ti,ab,kw OR 'drug complication*':ti,ab,kw OR 'high risk medication*':ti,ab,kw OR 'high risk drug*':ti,ab,kw) AND (antihypertensive*:ti,ab,kw OR 'blood pressure medication*':ti,ab,kw OR 'ace inhibitor*':ti,ab,kw OR arb*:ti,ab,kw OR 'angiotensin receptor blocker*':ti,ab,kw OR 'beta blocker*':ti,ab,kw OR 'calcium channel blocker*':ti,ab,kw OR 'alpha blocker*':ti,ab,kw OR diuretic*:ti,ab,kw OR 'angiotensin-converting enzyme inhibitor*':ti,ab,kw OR 'adrenergic blocker*':ti,ab,kw)

Query for Cochrane Library CENTRAL (89)	
	(pharmacogenomic* OR pharmacogenetic* OR pharmacogene* OR (genetic NEXT polymorphism*) OR (genetic NEXT variant*) OR (gene NEXT drug NEXT interaction*) OR (drug NEXT response NEXT gene*) OR (pharmacokinetic NEXT polymorphism*) OR (pharmacodynamic NEXT variant*)) in Title Abstract Keyword AND ((adverse NEXT drug NEXT reaction*) OR ADR OR (adverse NEXT drug NEXT event*) OR ADE OR (adverse NEXT drug NEXT effect*) OR (side NEXT effect*) OR (adverse NEXT effect*) OR (drug NEXT toxicity) OR (treatment NEXT intolerance) OR pharmacovigilance OR (drug NEXT related NEXT side NEXT effect*) OR (drug NEXT related NEXT adverse NEXT reaction*) OR (medicine NEXT side NEXT effect*) OR (ADR NEXT prediction) OR (adverse NEXT drug NEXT reaction NEXT prediction) OR (adverse NEXT outcome*) OR (undesirable NEXT effect*) OR (undesirable NEXT event*) OR (treatment NEXT emergent) OR (drug NEXT complication*) OR (high NEXT risk NEXT medication*) OR (high NEXT risk NEXT drug*)) in Title Abstract Keyword AND (antihypertensive* OR (blood NEXT pressure NEXT medication*) OR (ACE NEXT inhibitor*) OR ARB* OR (angiotensin NEXT receptor NEXT blocker*) OR (beta NEXT blocker*) OR (calcium NEXT channel NEXT blocker*) OR (adrenergic NEXT blocker*) OR (alpha NEXT blocker*) OR diuretic* OR (angiotensin NEXT converting NEXT enzyme NEXT inhibitor*) OR (adrenergic NEXT blocker*)) in Title Abstract Keyword - (Word variations have been searched)

Table S3: Ancestry-Stratified Population Allele Frequencies (AF)

Gene	rsID	Global AF	European AF (non-Finnish)	South Asian AF	East Asian AF	African AF
BDKRB2	rs8012552	0.53	0.58	0.51	0.51	0.45
MME	rs2016848	0.39	0.38	0.55	0.46	0.35
PTGER3	rs11209716	0.36	0.38	0.26	0.29	0.35
CYP3A5	rs15524	0.12	0.07	0.27	0.28	0.59
CYP3A5	rs4646453	0.04	0.02	0.22	0.24	0.07
CYP3A5	rs776746	0.88	0.93	0.72	0.73	0.29

Table S4: PRISMA 2020 checklist

Section and Topic	Item #	Checklist item	Location where item is reported
**TITLE**			
Title	1	Identify the report as a systematic review.	title
**ABSTRACT**			
Abstract	2	See the PRISMA 2020 for Abstracts checklist.	abstract
**INTRODUCTION**			

Section and Topic	Item #	Checklist item	Location where item is reported
Rationale	3	Describe the rationale for the review in the context of existing knowledge.	introduction
Objectives	4	Provide an explicit statement of the objective(s) or question(s) the review addresses.	introduction
**METHODS**			
Eligibility criteria	5	Specify the inclusion and exclusion criteria for the review and how studies were grouped for the syntheses.	methods
Information sources	6	Specify all databases, registers, websites, organisations, reference lists and other sources searched or consulted to identify studies. Specify the date when each source was last searched or consulted.	methods
Search strategy	7	Present the full search strategies for all databases, registers and websites, including any filters and limits used.	methods
Selection process	8	Specify the methods used to decide whether a study met the inclusion criteria of the review, including how many reviewers screened each record and each report retrieved, whether they worked independently, and if applicable, details of automation tools used in the process.	methods
Data collection process	9	Specify the methods used to collect data from reports, including how many reviewers collected data from each report, whether they worked independently, any processes for obtaining or confirming data from study investigators, and if applicable, details of automation tools used in the process.	methods
Data items	10a	List and define all outcomes for which data were sought. Specify whether all results that were compatible with each outcome domain in each study were sought (e.g. for all measures, time points, analyses), and if not, the methods used to decide which results to collect.	methods
	10b	List and define all other variables for which data were sought (e.g. participant and intervention characteristics, funding sources). Describe any assumptions made about any missing or unclear information.	NA
Study risk of bias assessment	11	Specify the methods used to assess risk of bias in the included studies, including details of the tool(s) used, how many reviewers assessed each study and whether they worked independently, and if applicable, details of automation tools used in the process.	methods
Effect measures	12	Specify for each outcome the effect measure(s) (e.g. risk ratio, mean difference) used in the synthesis or presentation of results.	NA
Synthesis methods	13a	Describe the processes used to decide which studies were eligible for each synthesis (e.g. tabulating the study intervention characteristics and comparing against the planned groups for each synthesis (item #5)).	methods
	13b	Describe any methods required to prepare the data for presentation or synthesis, such as handling of missing summary statistics, or data conversions.	NA
	13c	Describe any methods used to tabulate or visually display results of individual studies and syntheses.	methods
	13d	Describe any methods used to synthesize results and provide a rationale for the choice(s). If meta-analysis was performed, describe the model(s), method(s) to identify the presence and extent of statistical heterogeneity, and software package(s) used.	NA
	13e	Describe any methods used to explore possible causes of heterogeneity among study results (e.g. subgroup analysis, meta-regression).	NA
	13f	Describe any sensitivity analyses conducted to assess robustness of the synthesized results.	NA
Reporting bias assessment	14	Describe any methods used to assess risk of bias due to missing results in a synthesis (arising from reporting biases).	NA
Certainty assessment	15	Describe any methods used to assess certainty (or confidence) in the body of evidence for an outcome.	NA
**RESULTS**			
Study selection	16a	Describe the results of the search and selection process, from the number of records identified in the search to the number of studies included in the review, ideally using a flow diagram.	results
	16b	Cite studies that might appear to meet the inclusion criteria, but which were excluded, and explain why they were excluded.	supplementary
Study characteristics	17	Cite each included study and present its characteristics.	results

Section and Topic	Item #	Checklist item	Location where item is reported
Risk of bias in studies	18	Present assessments of risk of bias for each included study.	results
Results of individual studies	19	For all outcomes, present, for each study: (a) summary statistics for each group (where appropriate) and (b) an effect estimate and its precision (e.g. confidence/credible interval), ideally using structured tables or plots.	NA
Results of syntheses	20a	For each synthesis, briefly summarise the characteristics and risk of bias among contributing studies.	results
	20b	Present results of all statistical syntheses conducted. If meta-analysis was done, present for each the summary estimate and its precision (e.g. confidence/credible interval) and measures of statistical heterogeneity. If comparing groups, describe the direction of the effect.	NA
	20c	Present results of all investigations of possible causes of heterogeneity among study results.	NA
	20d	Present results of all sensitivity analyses conducted to assess the robustness of the synthesized results.	NA
Reporting biases	21	Present assessments of risk of bias due to missing results (arising from reporting biases) for each synthesis assessed.	NA
Certainty of evidence	22	Present assessments of certainty (or confidence) in the body of evidence for each outcome assessed.	NA
**DISCUSSION**			
Discussion	23a	Provide a general interpretation of the results in the context of other evidence.	discussion
	23b	Discuss any limitations of the evidence included in the review.	discussion
	23c	Discuss any limitations of the review processes used.	discussion
	23d	Discuss implications of the results for practice, policy, and future research.	discussion
**OTHER INFORMATION**			
Registration and protocol	24a	Provide registration information for the review, including register name and registration number, or state that the review was not registered.	Abstract, Methods and PROSPERO
	24b	Indicate where the review protocol can be accessed, or state that a protocol was not prepared.	Abstract, Methods and PROSPERO
	24c	Describe and explain any amendments to information provided at registration or in the protocol.	NA
Support	25	Describe sources of financial or non-financial support for the review, and the role of the funders or sponsors in the review.	Funding support- manuscript
Competing interests	26	Declare any competing interests of review authors.	manuscript
Availability of data, code and other materials	27	Report which of the following are publicly available and where they can be found: template data collection forms; data extracted from included studies; data used for all analyses; analytic code; any other materials used in the review.	manuscript

From: Page MJ, McKenzie JE, Bossuyt PM, Boutron I, Hoffmann TC, Mulrow CD, et al. The PRISMA 2020 statement: an updated guideline for reporting systematic reviews. BMJ 2021;372:n71. doi: 10.1136/bmj.n71. This work is licensed under CC BY 4.0. To view a copy of this license, visit https://creativecommons.org/licenses/by/4.0/
